# Community based intervention to prevent domestic violence against women in the reproductive age in Northwestern Ethiopia: a protocol for quasi-experimental study

**DOI:** 10.1186/s12978-017-0414-2

**Published:** 2017-11-21

**Authors:** Agumasie Semahegn, Kwasi Torpey, Abubakar Manu, Nega Assefa, Augustine Ankomah

**Affiliations:** 1 Department of Population, Family and Reproductive Health, School of Public Health, College of Health Science, University of Ghana, Legon, Accra Ghana; 2 College of Health and Medical Sciences, Haramaya University, Po. Box 235, Harar, Ethiopia

**Keywords:** Domestic violence, Women, Intervention implementation, Community, Ethiopia

## Abstract

**Background:**

Violence against women is a well understood devastating global pandemic, and human right violation. One in three women experienced intimate partner violence worldwide. In Ethiopia, the level of domestic violence against women is one of the highest in the world. However, Ethiopia is signatory for various conventions and incorporated in legal frameworks. Nevertheless, effective implementation of the existing policy documents, and engaging different stakeholders is very limited. Therefore, we aimed to pilot feasibility of implementing available research evidence and policy documents at community level to prevent domestic violence against women in Awi zone, northwestern Ethiopia.

**Methods:**

A community-based quasi-experimental study design will be employed using mixed method. Multistage stratified systematic sampling and purposive sampling will be used to recruit quantitative and qualitative study participants, respectively. A total of 1,269 women will be participated in the intervention, active comparator and control groups. Pre and post-test quantitative data will be collected using face-to-face interview. Qualitative data will be collected through in-depth, key informant interview and focus group discussions. Intervention: advocacy meeting will be held to persuade local politicians and sustain the implementation of community based intervention to prevent domestic violence against women. Community representatives will be trained to enhance peer education to promote community awareness and engage stakeholders to transform the traditional gender norm within local context. Awareness creation and husband involvement will be made through integrating the intervention with community health extension program. Only husband involvement will not be promoted in the active comparator to test the role of husband involvement on the domestic violence prevention activities. Intervention progress will be monitored regularly. Gathered data will be entered in Epidata and exported to SPSS (23.0) software for analysis. Descriptive statistics, logistic regressions, intention to treat analysis and difference in differences will be computed. Qualitative data will be transcribed, color coded, thematically analyzed and arranged using Nvivo.

**Discussion:**

This interventional study is aimed to design, pilot and translate proven research evidence, agreed conventions and country policy document to real setting implementation. We are expecting to initiate implementation of culturally acceptable intervention through engaging stakeholders. Policy makers, planners and any concerned bodies will be benefited.

**Trial registration:**

ClinicalTrials.gov ID: NCT03265626

## Plain English summary

Violence against women is a well-recognized reproductive health issue. It is a cross-cutting issue which is interlinked with every day’s routine activity and overall development agenda in the world. Several primary studies have been conducted on the area that briefly report the level and associated factors of the domestic violence against women. Moreover, some interventional studies also conducted in different parts of the world revealed that interventions mainly focused on gender norm, societal culture and other behavioral change communication can improve the prevention strategies to tackle domestic violence against women. Nevertheless, many countries still have a limited motivation to implement their policy in real setting to prevent violence against women. One of the most dilemmatic issue is considering violence against women as a private matter, and still there is lack of clear understanding on the difference of private and public matters. Therefore, this study aims to mobilize community to prevent domestic violence against women. It will contribute evidence to program planners, policy makers, clinicians and other stakeholders to have informed decision on the issue of domestic violence against women.

## Background

Gender based violence against women is a well-recognized public health problem, gross pervasive abuse of human rights, and major obstacle for achieving the sustainable development goals worldwide [1–6]. Globally, one in three women experience violence against women (VAW) [4, 7, 8]. About 38–60% of murders of women are committed by intimate partners every year. Domestic VAW is not only an exhibit of unbalance of power, and a bad women’s life experience at their home [9, 10], it accounts for 5% loss of women’s healthy years. The morbidity and mortality due to VAW is more than a cumulative burden of cancer, road traffic accidents and malaria, a huge threat and unmanageable global burden to public health for the future [10]. According to World Health Organization (WHO) multicounty study indicated that intimate partner violence in Ethiopia was 71%, which is the highest in the world [11]. Research evidence show that domestic violence is strongly linked with gender inequality that affect women’s ability to have a discussion on several reproductive health issues including human immunodeficiency virus (HIV) and negotiate on condom use [12–14]. The cost of VAW is estimated from 1.2–3.7% of the country’s gross domestic product, which is equivalent to what many governments spend on primary education [8]. Nevertheless, most governments have considered domestic VAW as minor social tricky, private matter and not taken as crime [1, 10, 12].

The United Nations aims to create an enabling household environment, improve women’s right, political, economic empowerment and legal protection [15]. Consistently, the Maputo Plan of Action (2016–2030) gives due attention to the implementation of international, regional and national legislations to tackle VAW [16]. On the other hand, a systematic review suggested that women empowerment and community mobilization averted $13–19 per disability adjusted life year. Hence, gender responsive interventions that target traditional gender norms, behavior change communication and structural drivers are cost effective approaches to prevent VAW [17]. Likewise, domestic VAW is strongly associated with gender norms, poverty, denied access to education, embedded in social customs, lack of autonomy, inequitable gender attitudes, women accept justified wife beating and alcohol use [1, 2, 5, 18–23].

**Fig. 1:**
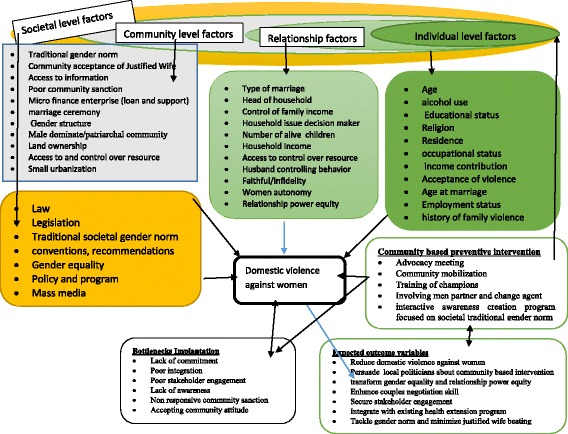
Conceptual framework adapted from ecological model

Moreover, domestic VAW is significantly associated with various poor health outcomes (low birth weight, premature birth, malnutrition, suicide, homicide, mental illness, physical injuries, disability and non-communicable diseases) [1, 6, 7, 18, 20, 24–29]. In addition, VAW has been associated with HIV and other sexual transmitted infections (STIs) acquisition, unintended pregnancy, induced abortion and other poor reproductive health conditions [1, 5, 7, 10, 14, 19–21, 23, 27, 30–43]. It is a cross cutting issue which requires comprehensive approach to address it (microfinance support, equality norm or culture transformative training; communication skill in relationship; minimize access to alcohol use and community health education [1, 20, 44]. The conceptual framework that shows the detail of factors associated with domestic VAW included as an additional file (Fig. 1).

In Ethiopia, domestic VAW is a common women’s life experience. A systematic review on studies done in Ethiopia (2000–2014) indicated that any intimate partner violence (IPV) is a known phenomenon in the country which ranged from 20 to 78% [45]. In response to this, the government of Ethiopia has incorporated the issue of women’s right and gender equality in the constitution (Article 35 and 89(7)) [46], criminal code under proclamation NO.414/2004 *(Article 564*) [47] and Family Code Proclamation No. 213/2000 [48]. Violence against a marriage partner or a person cohabiting in an irregular union is prohibited. In addition, access to and control over resource including other rights and responsibilities of partners in marriage or cohabitation are clearly addressed in the policy documents. Moreover, Ethiopian Ministry of Health has developed a standard operating procedure for the response and prevention of sexual violence in Ethiopia in 2016 [49]. Community mobilization, stakeholder engagement and partner involvement are the most prioritized intervention programs which need effective implementation, but these are still very limited. Furthermore, this implementation research will give an insight to mobilize resource, involve stakeholders to put the existing law and regulations into action and identify implementation bottlenecks. Given the paucity of evidence, it will provide information on feasibility and implementation of community based preventive intervention programs.

### Study objective

The overall aim of this study is to assess the outcome of community based intervention to prevent domestic violence against married or cohabiting women in the reproductive age and associated factors in Awi zone of the Northwestern Ethiopia from November 15, 2017 to November 15, 2018**.**

### Specific objectives

1. To assess the baseline level of domestic VAW in the study area
2. To identify associated factors of domestic VAW in the study area
3. To explore implementation bottlenecks in VAW program in the study area
4. To implement culturally acceptable interventions to address domestic VAW
5. To assess the outcome of community based intervention on domestic VAW

## Methods

### Study setting

This study will be conducted in Awi zone, Amhara regional state, Northwestern Ethiopia. Enjibara town is the administration center of Awi zone which is located 447 km from Addis Ababa. Based on the 2007 Census, it has a total population of 982,942 and 491,077 of them are women. Only 12.5% of population are based in urban. Based on the preliminary assessment; Awi zone administration has nine districts with a total of 204 sub-districts and 6463 development armies. *Sub-district is the smallest administrative unit which has an estimated 1000–1500 households or 5000 total population*. In one of the district of the Awi zone, Faggetalekoma district has one of the highest record in domestic VAW [45, 50]. The women and children affair office has structured by the government to execute such type of issues. The zonal health department and district health offices have been implementing the standard operating procedures to respond and prevent sexual violence.

### Study design and participants selection

Community based three arm quasi-experimental design will be employed usingquantitative and qualitative techniques (mixed methods).

### Quantitative study participants

**Fig. 2:**
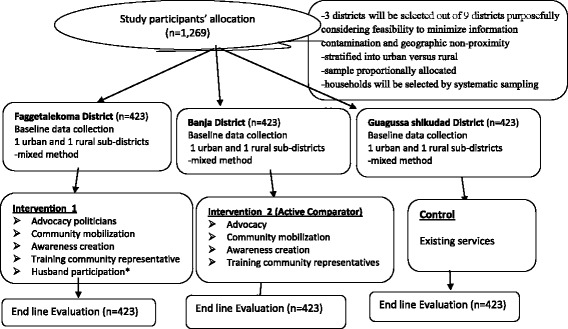
Illustration of quasi-experimental design for intervention implementation

Married or cohabiting women (15–49 years) will be recruited for the quantitative study. Study participants will be allocated into intervention, active comparator and control groups. Three districts will be selected out of nine districts considering stability or mobility status, convenience to implement intervention and monitor the control. The study design and participant allocation is illustrated as an additional file (see Fig. 2).

### Qualitative study participants

Community representatives (local leaders, religious leaders and elders), husbands, health care providers, police and women affair office representatives will be involved.

### Inclusion criteria

- Women (15–49 years) from the general population
- Married/cohabited women who have stayed at least 12 months with the current husband
- Women and community representatives who have registered or recognized as permanent resident at least for 6 months

### Exclusion criteria

- Women and or community representatives who will unable to respond due to severe physical or mental illness

### Sample size determination

The sample size is determined considering both the intervention and control using a formula recommended for dichotomous outcome variable. We assumed that implementation of community based intervention program to prevent domestic VAW is superior as compared with the standard service [51]. In addition, we considered 5% margin of error, 95% significance level and 80% power.

$$ N=2\times {\left(\frac{z_{1-\alpha }+{z}_{1-\beta }}{\mathrm{d}\hbox{-} {\delta}_0}\right)}^2\times p\times \left(1-p\right) $$

Where as; N is the sample size of each group, P is prevalence in standard intervention (control arm), d: the real difference between two treatment effects (d = P_o_-P_x_); 22%–9% = 13% = 0.13: study done in Uganda (22%) in the standard intervention group [52]; δ_0:_ statistical acceptable margin (5%); ɑ: significance level at 95% (ɑ = 1–0.95 = 0.05 = 1.645), ß_:_ 80% power (ß = 1–0.8 = 0.2 = 0.845. Then, 15% is added for potential non-achievements. Furthermore, due to the multistage sampling, design effect is considered to boost the power and precision that taken from the design effect of women experienced physical or sexual violence in the study area (Amhara region) in the last 12 months was 1.11 [53]. Eventually, a total 1269 women is obtained for the three arms (i.e. 423 for each group). The number of qualitative study participants will be determined by information saturation. However, a minimum 12 in-depth interviews and two focus group discussions (FGDs) (male versus female groups) which comprise 8–10 discussants will be conducted.

### Sampling procedure

Multistage, stratified and systematic sampling will be used to recruit study participants at their permanent place of residence. Three districts are selected out of nine districts in Awi zone (comprehensive intervention, active comparison and control). Then, six sub-districts (two sub-districts from each district) will be selected purposefully considering appropriateness for intervention, resource, and geographical non-proximity to control information contamination between intervention and controls. Then, sub-districts will be stratified (urban versus rural) to control the awareness level, access to information and community traditional gender norm variability between urban and rural dwellers. After that, the sample of the group will be proportionally allocated to size of each selected sub-district. The number of eligible households for this study will be identified using the community based health extension workers registry. Eventually, systematic sampling will be employed to select actual study participants at household level. Unique code will be given to selected households for intervention monitoring and finally endline evaluation purpose. In the meantime, if two married women are present in one household, only one woman will be selected using lottery method for interview.

Purposive sampling method will be used to select study participants for in-depth interview, key informant interview (KII) and FGDs. Community leaders will be consulted and actively involved on the recruitment of the most knowledgeable community representatives for in-depth interview, KII and FGDs. It will be conducted to explore individual and community perception about domestic VAW, sex negotiation, decision making, community gender norm, wife beating attitudes and community responses.

### Measurement tools

Data will be collected using structured and semi-structured questionnaire that is adapted from various literature [29, 41, 50, 54–70]. The tool will be pretested and modified to suit with the local context. It will consist of demographic, socio-economic variables about the women, relationship, community and societal level factors of domestic violence. In addition, the tool will comprise questions about the experience of psychological, physical and sexual violence. Moreover, semi-structured interview guide will be designed for qualitative study to explore the underlying bottlenecks in the community that hinder intervention implementation.

### Data collection procedure

**Fig. 3:**
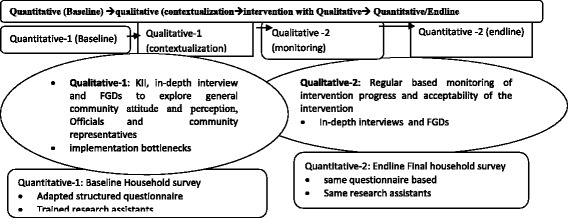
diagrammatic show of the sequential explanatory mixed-method data collection process

Data will be collected using sequential explanatory mixed method (quantitative 1 and 2, and qualitative 1 and 2) for baseline, monitoring and endline assessment. It is illustrated and attached as additional file (see Fig. 3) that is adapted from other study [71]. Face-to-face interview will be used for quantitative data collection. In-depth interview, KII and FGDs will be carried-out for qualitative study. The KII will be conducted on community representatives, district council members, women affair office, police officers, lawyers, health care providers and nongovernmental organization. Gender specific FGDs will be held on men and women to assess societal perception about domestic VAW. All participants’ socio-demographic data will be captured using anonymously coded structured questionnaire. Voice recorder, note taking and non-verbal communication during the discussion will be recorded as much as possible. An estimate of 60 to 90 min will be taken to conduct one FGD. Eventually, endline evaluation will be carried-out using the same structured questionnaire, data collectors and supervisors as of the formative assessment. Twelve female nurses, midwives or public health officers will be recruited to collect the data.

#### Data quality assurance activities

- Participants Allocation: Intervention and control group participants will be allocated based on their geographic non-proximity to minimize information contamination. In addition, regular monitoring will be carried out on both intervention and control groups.
- Data Collector’s Recruitment and Training: Data collectors will be recruited through consulting local leaders to get appropriate data collectors. Then, female nurses, midwives and or public health officers who have diploma or above qualification will be recruited as data collector (research assistant) to enhance quality of the data to be collected. Two supervisor who have Master of Public Health and had previous field experience will be recruited. Three days training will be given for research assistants by the principal investigator which will mainly focus on study objectives, sampling methods, data collection tool and procedures, interview techniques and ethics. Biomedical research ethical principles and WHO gender based violence research ethical guideline will be explained to research assistants. Regular supportive supervision will be given by principal investigator and supervisors.
- Implementers Recruitment and Training: Four community health extension workers as implementer and one supervisor will be recruited and deployed during the entire intervention period. Health extension workers who have been working in the sub-district will recruited as intervention implementer to ease integration with the existing innovative community based health extension program and working very closely with the community. Health Extension Workers (HEWs) will be re-oriented on the objectives, domestic VAW, gender inequality norm and how to integrate with their health extension package. Regular discussion and close supportive supervision will be given by principal investigator and facilitators.
- Pretest questionnaire: Pretest will be done on 5% of the sample size from nearby district to test sensitive words in the questionnaire, sequence of questions, sampling, data collection procedure, consent taking, interview skill and note taking. Necessary amendment will be made accordingly.
- Tool Development and Validation: Intervention materials will be adapted from WHO and United Nation Population Fund (UNFPA) materials, research evidence, human right protection instrument and national policy documents. Most of domestic VAW research findings recommend addressing of wife beating accepting attitude, gender norm, gender equality, women empowerment, male involvement and legal sanctions. The adapted intervention materials for community mobilization, awareness creation and training of community representatives will be peer reviewed for validation. The research team will review it and necessary modification will be made accordingly. In addition, any feedback about the material will be received from advocacy meeting participants to contextualize with the community setting.

### Data processing and statistical analysis

Both baseline and endline data will be checked and edited for completeness and consistency at field as well as at office. Explanatory and outcome variables will be clearly predefined prior to data entry, in which domestic VAW will be coded as if a woman reports at least one type of experience of either physical, psychological or sexual violence coded as “Yes = 1” and “No = 0”. Similar coding will be done for physical, sexual and psychological domestic violence against women to carry out subgroup analysis. On the other hand, women’s wife beating accepting attitude will be measured using six questions or measurements, and also if women respond at least one justifying wife beating [58, 59, 61]. Similarly women autonomy will be measured in four indicators such as economic decision-making, reproductive health service utilization decision-making, extent of freedom of movement, and women’s attitudes toward violence [54].

Then, data will be entered in Epidata (3.5.1) and exported to SPSS (23.0) for further cleaning and analysis. Multiple imputation will be done to handle missing data as necessary. Descriptive statistics, binary and multiple logistic regressions and Intention to Treat Analysis (ITTA) will be computed. Moreover, Difference in Differences (DID) will be calculated to determine the net intervention effect. Finally, statistical significance of intervention effect will be measured using adjusted prevalence ratio, adjusted odds ratio/risk ratio at 95% confidence interval (CI) and *P*-Value less than 0.05. On the other hand, qualitative data will be transcribed through playing and replaying the voice recorder and also narrating the note taken during interviews or FGDs. The voice recorder audio will be listen as much as possible in quiet place. It will be transcribed independently by research assistant and principal investigator according to the verbatim of participants'. Then transcribed qualitative data will be translated from local language (Amharic) to English. The quotes will be narrated and cited using participant’s code. Thematic areas will be identified and arranged based on their themes. Ideas will be color coded, and then merged accordingly. Qualitative data will be analyzed or arranged using Nvivo software.

### Implementation Design of the Community Based Intervention

Evidence from clustered randomized controlled trials, community based quasi-experimental trials, longitudinal studies and systematic review on domestic VAW have been used to design intervention. In addition, recommendation of various community based research evidence, WHO recommendations, existing legal sanctions or policy documents (constitution, civil code, family laws and standard operation procedure to respond and prevent domestic VAW) will be used to implement the intervention. Intervention will be implemented using culturally appropriate and integrating with existing innovative community based health extension program. Interventions will be more focused on traditional gender norm, negotiation skills and involve men on domestic violence prevention activities. The intervention implementation will have contextualization, tool development and validation, advocacy, stakeholders’ engagement and overall community mobilization activities.

### Advocacy meeting and stakeholders engagement

Advocacy meeting will be held to enhance awareness about domestic VAW problem, socioeconomic consequences, political implications, evidence based prevention strategies and country legal sanctions towards VAW. The advocacy meeting will be held every three months regularly for a year. It will create an entry point into the community and also involve stakeholders. Both the zonal and district health offices, women and child affair offices, police and security, office of justice, agriculture and land management office, women lawyer associations, nongovernmental organizations, community representatives (religious fathers, local leaders and elders) will be considered as potential stakeholders. However, stakeholder analysis will be made in Awi zone perspective to identify key stakeholders. Then, identified key stakeholders will be expected to be actively involved on the implementation process as per their hierarchy. Gender expert from health office and women and children affair office will be actively involved in the intervention implementation process.

**Fig. 4:**
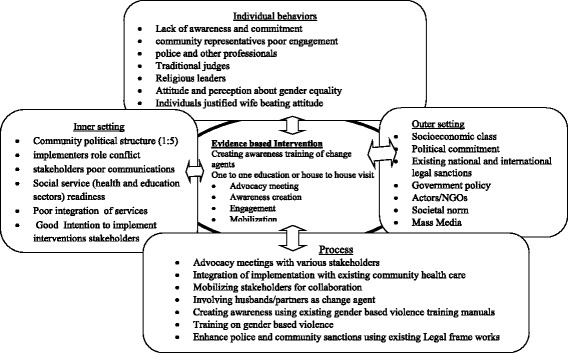
Schematic presentation of Interacting domains using CFIR

The intervention will be implemented to address the risk factors according to Ecological Model (individual, relationship, community and societal level factors). Furthermore, piloting implementation intervention will focus on the five domain of implementation research domains (intervention, inner, outer, individual behavior and process settings) which clearly illustrated on Consolidated Framework for Implementation Research (CFIR) model, and presented in detail as additional file (see Fig. 4).

Arm 1: Comprehensive intervention group: comprehensive intervention to prevent domestic VAW and enhance existing policies will be conducted. The intervention will be focused on physical, psychological and sexual violence, decision making, negotiation, communication on household matters, gender equality and equity norms, and their relationship with women’s health. Gender based training will be addressed about transformative gender norm. Comprehensive intervention will be comprised of the advocacy meeting, community mobilization activities, husband involvement and other awareness creation activities.

- Advocacy meeting/workshop: will be made to convince local people who are in position (authority) (police, women association, women affair office, health professionals) that have direct contribution to tackle VAW. Research evidence that show the magnitude, associated factors and proven solutions will be presented by the principal investigator. Thorough discussions will be made on the issue of VAW as per the local setting. It will allow to convince as well as dig out implementation bottlenecks. Leaflets, posters and any other awareness creation materials will be distributed to improve community attitude about VAW.
- Community mobilization: will be done at different public places (church and other social gathering events) about harmfulness of VAW, gender equality and equity, women autonomy on reproductive health, decision making on household issues and negotiation/communication skill. In addition, awareness creation activities will be carried out as community conversation to reach the entire community within the study sites.
- Training: will be provided to community representatives as a change agent (leaders, religious fathers, elders, health care provider, police, local politicians and associations). Community believes, culture and religion are the main factors that affect attitude and gender norms. The training aims to improve awareness of public figures towards gender norm specifically on VAW. On each session of the training, discussion will be entertained to have common understanding of issues and discuss possible solutions. All trainees are expected to disseminate information while going back to their home or religious place, community gathering areas (*Idir, Equib,* etc.) and also Sunday coffee ceremonies. Regular trainings to community representatives will be given every three months for a year in each intervention group.
- Awareness creation information dissemination: well oriented health extension workers will implement the awareness creation activities through house-to-house by integrating with the health extension package. The awareness creation will be more focused on gender equality, equity, decision making autonomy, negotiations skill in relationship and etc. this intervention will be given mainly to the women.
- Husband or partner involvement on domestic violence prevention: group health education and discussions will be held with husband or partners. Gender equality, equity, decision making, communication and involving on reproductive health issues as part of human right are the main issues that will be mentioned in this target group. Women’s contribution in development and their right involving on household issues decision making will be raised and discussed.

ARM 2: Active comparator group: are all interventions listed above under comprehensive intervention (advocacy, community mobilization, awareness creation and training community representatives) will be provided to this group except active husband involvement intervention. The ultimate objective is to test the contribution of husband involvement on domestic VAW prevention.

Arm 3: Standard services (control group): the standard service will be sustained. We will regularly monitor to know what is going on in addition to the standard service during the study period for documentation purpose only.

#### Expected outcomes

**Table:** 

Expected Outcomes of the Community Based Intervention	
Specific objective 1	Conduct baseline assessment of domestic VAW in the study area
	- determine the prevalence of domestic VAW (physical, sexual, psychological)
Specific objective 2	Identify associated factors of domestic VAW
	- Identify the associated factors - explore community perception - Explore stakeholders engagement level - explore implementation barriers or bottlenecks of VAW program at implementers side
Specific objective 3	Design intervention implementation strategy to tackle domestic VAW
	- Prepare the intervention material and validate according to the local culture - Integrate with community based health extension program - Conduct advocacy workshop and work with the local administrative bodies
Specific objective 4	Implement culturally acceptable interventions to address bottlenecks and DVAW
	*advocacy workshop four times a year (4 session)
	- create awareness on politicians - create champions of domestic VAW prevention - enhance commitment to implement policy documents at community level
	*community mobilization monthly for a year (12 sessions)
	- involving the community in the domestic VAW prevention - engaging stakeholders - create community ownership
	*Awareness creation to the women (at least once per month)
	- enhance their communication and negotiation skill - enhance their awareness about gender equality - transform their wife-beating acceptance attitude
	*involving husbands (6 to 12 sessions)
	- participate husbands/partner as change agent - enhance their awareness about gender equality - improve masculine attitude and transform
Specific objective 5	To assess the outcome of community based intervention on domestic VAW
	- conduct endline assessment - perform data analysis to determine the prevalence of domestic VAW - compute the difference between endline and baseline (prevalence, community acceptance, factors) - integrate with health extension program - scale up to other sites - learn from success and failure

**Table:** 

Phase 1: Baseline	Phase 2: intervention implementation	Phase 3: endline	Phase 4: dissemination and scale up
-Baseline data collection -Instrument validation -Contextualization of the intervention -explore community perceptions	-advocacy meeting with local administrative bodies and stakeholders to explore implementation barriers and also enhance engagement of stakeholders -community mobilization -awareness creation to the women on gender equality, rights and community norm -awareness creation and enhance husband/partner involvement -Training of community representatives to involve them in the traditional norm change agent -traditional coffee ceremonies and community conversation	-endline data collection - statistical analysis to investigate the intervention effect between baseline and endline -identify barriers and enablers for the future domestic violence prevention	*Dissemination
			-Advocacy meeting -policy Brief -publication in peer review journals -local Media -use social media -present in conference s
			*Scale up of successful experiences
			-learn from barriers and propose alternative solutions -integrate with health extension program -engaging stakeholders/ community
M& E	M& E	M& E	
-measurement tool and intervention material validation -number of women interviewed per day and completeness and consistency of questionnaire	-number of advocacy workshop -pre-posttest of the participant -feedback from participants -number of women involve in awareness creation -number of community representatives trained and involve on community mobilization -number of husbands/involved and attend awareness creation program - implementers briefing and debriefing regular session -KII and FGD on the progress of the intervention and acceptability of the intervention by the community	-re-orientation of data collectors -use same data collectors and same site -using same household and make sure that the women is the former participant -determine prevalence of domestic VAW -identify barriers -determine difference in difference -note that; the control group will be monitored regularly on what is going on in addition to the standard care and findings will be documented using reporting checklist.	

#### Phase of intervention and monitoring and evaluation (M&E)

## Discussion

This study will pilot the feasibility and effectiveness of the community based intervention to prevent domestic VAW in the northwestern Ethiopia. Interventional studies conducted in Uganda indicated that community-based intervention successfully avert VAW and reduced unnecessary spending on related costs [72, 73], On the other hand, a study done in South Africa revealed that integrated alcohol and HIV prevention intervention reduced VAW by 70%, and also the gender based violence with HIV prevention intervention had reduced significantly the intention of perpetration by 50% [74]. Another study in Ethiopia showed that combined intervention (community engagement with group education) reduced IPV by 60% [75]. Furthermore, microfinance based intervention brought significant change in three African countries. A study done in Uganda shows that microenterprise as economic empowerment to prevent IPV brought significant improvement on income, women perception on their autonomy, control behaviors and quality of marital relationship [76]. Studies in Côte d’Ivoire (2010–2012) revealed that economic VAW reduced by 61% [77, 78]. In addition, financial support to promote safe sex, balance relationship power and transform couple’s attitude in Tanzania reduced VAW by 30%. Remarkably, VAW also reduced by 57% in couples who had equal power [79].

Likewise, a quasi-experimental study employed in Tanzania (2012) confirmed that community based intervention using local mass media and advocacy meeting improve knowledge on sexual VAW significantly from 57.3 to 80.6%. The combined intervention had a substantial effect on awareness raising and improve attitude towards VAW norms (*p* < 0.001) [80]. Therefore, evidence from randomized controlled trail, quasi-experimental and longitudinal studies concluded that community based comprehensive intervention such as integrated IPV prevention services (economic empowerment, gender norm transformation to promote gender equality and equitable relationship power, involving men, mass media and advocacy meeting) are some of proven evidences based interventions to prevent IPV. These intervention could tremendously reduce IPV [74–76, 79, 81, 82]. Primary prevention strategies (combined microfinance with addressing gender equality norm or culture transformative training; communication and relationship skills; minimize access to alcohol use, sensitization, health education and other services) are recommended by WHO to prevent VAW [20]. Similarly, the government of Ethiopia has developed a standard operation procedure for responding and prevention sexual VAW, and clearly stated the implementation to the real setting is very limited [49]. Therefore, this piloting feasibility and effectiveness study will address bottlenecks, design community based intervention strategies and mobilize the community effort to tackle domestic VAW.

### Plan for dissemination of research findings

At the end of the interventional study, a study finding will be compiled. The finding will be disseminated to key stakeholders involving in the study. Scale upping and follow up will be made to ensure the sustainability of the intervention to prevent domestic VAW. The principal investigator will take the leading role of the finding dissemination activities. Short communications (poster, policy brief, abstract presentations) will be carried out. Efforts will be made to publish the finding in international peer reviewed and reputed journals.

## Abbreviations

CEDAW: Convention on Elimination of any Discriminations against Women
CFIR: Consolidated Framework for Implementation Research
CI: Confidence Interval
DID: Difference in Differences
FGD: Focus Group Discussion
HEW: Health Extension Worker
HIV: Human Immunodeficiency Virus
IPV: Intimate Partner Violence
ITTA: Intention to Treat Analysis
KII: Key informant Interview
M&E: Monitoring and Evaluation
NGO: Nongovernmental Organization
STI: Sexual Transmitted Infection
TDR: Tropical Disease Research
UNFPA: United Nations population Fund
VAW: Violence against Women
WHO: World Health Organization
